# A Wearable Inertial Measurement Unit for Long-Term Monitoring in the Dependency Care Area

**DOI:** 10.3390/s131014079

**Published:** 2013-10-18

**Authors:** Daniel Rodríguez-Martín, Carlos Pérez-López, Albert Samà, Joan Cabestany, Andreu Català

**Affiliations:** Technical Research Centre for Dependency Care and Autonomous Living–CETPD, Universitat Politècnica de Catalunya–Barcelona Tech, Rambla de l'Exposició 59-69, Vilanova i la Geltrú 08800, Barcelona, Spain; E-Mails: carlos.perez-lopez@upc.edu (C.P.-L.); albert.sama@upc.edu (A.S.); joan.cabestany@upc.edu (J.C.); andreu.catala@upc.edu (A.C.)

**Keywords:** inertial sensors, hardware, firmware, autonomy, accelerometry, Parkinson's disease

## Abstract

Human movement analysis is a field of wide interest since it enables the assessment of a large variety of variables related to quality of life. Human movement can be accurately evaluated through Inertial Measurement Units (IMU), which are wearable and comfortable devices with long battery life. The IMU's movement signals might be, on the one hand, stored in a digital support, in which an analysis is performed *a posteriori*. On the other hand, the signal analysis might take place in the same IMU at the same time as the signal acquisition through *online* classifiers. The new sensor system presented in this paper is designed for both collecting movement signals and analyzing them in real-time. This system is a flexible platform useful for collecting data via a triaxial accelerometer, a gyroscope and a magnetometer, with the possibility to incorporate other information sources in real-time. A μSD card can store all inertial data and a Bluetooth module is able to send information to other external devices and receive data from other sources. The system presented is being used in the real-time detection and analysis of Parkinson's disease symptoms, in gait analysis, and in a fall detection system.

## 1. Introduction

Recent technological developments in the automotive industry, telecommunications and electronics have enabled a major evolution in the possibilities of measuring and monitoring human movement [1]. One of the main current interests is analyzing human motion in fields such as sports [2] and health [3]. From the point of view of the field of dependency care, activity assessment and monitoring through human movement analysis is of great importance. It is applied to the evaluation of quality of life [4–6] and the ability of some individuals to walk [7]. Furthermore, human movement analysis is being used as a tool in for both treatment and clinical diagnosis [8,9].

Accelerometers and gyroscopes based on Micro-Electro-Mechanized-Systems (MEMS) technology have become the most used sensors in the study of human movement [10] because they are small, light, wearable and non-invasive [11]. These sensors are commonly used with a microcontroller able to process the measurements obtained. Moreover, peripherals such as a Bluetooth module are often included in order to enable communication with other devices. Systems made up from a combination of these capabilities have come to be called Inertial Measurement Units (IMU). These IMUs are now being widely used in human movement analysis [6,12–16] since they have been shown to be wearable and comfortable devices that can work autonomously for long periods.

IMUs have been employed for human movement analysis with several possible goals, such as measuring energy expenditure [17] or performance variables in sports [18]. In the dependency care field, they are employed in the assessment and monitoring of activities and symptoms since they enable the evaluation of the quality of life [4–6]. Attempts to monitor activities and symptoms rely on supervised learning methods. For example, within the field of studying Parkinson's disease (PD), decision trees [19], linear classifiers [20] or Support Vector Machines (SVM) [21–23] have been used to determine specific symptoms of the disease. Supervised learning methods were also employed in epilepsy episodes classification [24] and activity analysis in stroke patients [25]. This specific type of learning algorithm requires a training process that involves labeled data. In consequence, these research studies gathered and labeled signals in order to generate detection methods. That is to say that the IMU's were used as dataloggers. In this specific application of IMUs, signals are either collected and saved within a flash memory unit such as a μSD card or sent by means of either wireless or wired connections, such as Bluetooth or UART. The resulting supervised learning algorithms that determine symptoms or activities are referred in this paper as *offline* classifiers since they rely on previously collected data and they cannot provide real-time detection.

Offline classifiers can provide relevant information to clinics such as symptoms through the analysis of data previously captured and collected. In contrast, *online* classifiers provide relevant information in real-time; that is, concurrently to the IMU's signal acquisition. Online classifiers are of great interest. While offline classifiers enable the monitoring of a disease, online classifiers open the additional possibility of treating a disease. For instance, they have enabled PD patients to overcome Freezing of Gait (FoG) episodes by means of audio cues [26]. Moreover, online classifiers have been used in a clinical study to determine symptoms and motor states in PD patients [27,28]. This data was then employed to determine the amount of medication to be administered by the apomorphine infusion pumps used to avoid undesirable symptoms [21,28]. Similarly, insulin can be regulated in diabetic patients according to the current levels of glycemia determined through online classifiers [29,30]. Finally, online classifiers are used in patients with drop foot syndrome to correct their gait in real-time through Functional Electrical Stimulation (FES) [31].

In these different applications, online classifiers convert sensors signals into small pieces of relevant information whose storage requires less memory than the raw signals. For instance, several seconds of signals are converted into simple values that represent the presence of FoG, the motor state of a PD patient or the need to activate FES. Therefore, online classifiers enable IMU's to drastically reduce the information stored or transmitted. Although this approach increases microcontroller energy consumption, it enlarges the autonomy of the IMUs by eliminating the need to send or store raw data.

Summarizing the current technical hardware developments in the human movement analysis field, from the authors' point of view, there are two main applications of IMUs that can be found. On the one hand, IMUs may be used for monitoring purposes as datalogger devices. On the other hand, IMUs may be used in the real-time monitoring and treatment of a disease by including online classifiers in the IMU's microcontroller. To the best of our knowledge, there is no device available on the market, as will be shown in Section 3, able to satisfy both functionalities: collecting data for the creation of offline classifiers and allowing the development and implementation of online detectors. A device that offers both functionalities would require the IMU to provide a transparent and fast data acquisition to the online algorithms developed in order to allow their implementation into a structured code. Moreover, a flexible usage of the peripherals must be provided in order to debug the online classifiers. This double functionality is necessary to develop supervised learning algorithms and to use them in order to treat diseases. Thus, an IMU with the aim of satisfying both functionalities was developed and is presented in this work. Specific requirements that guided the IMU design are presented in Section 2. This device, called *9* × *2*, has been used in the clinical study in which PD symptoms were treated in real-time. This study is called Home-based Empowered Living for Parkinson's disease (HELP) [27] and recently won the award for best concluded 2013 European Project [32]. Previously, *9* × *2* was used as a datalogger in a national study called MoMoPa [33] in which movement signals from 35 PD patients were gathered. Recently, *9* × *2* has been used to collect movement signals in four different countries from 90 PD patients under the REMPARK project [34,35]. Finally, *9* × *2* is also being validated as part of a fall detector system in a pilot test with 200 individuals [36].

This paper is organized as follows: first, the desired requirements for the IMU are described. Next, current IMUs available as commercial products or research devices are presented. The following sections detail the IMU's hardware and firmware, respectively. Finally, some conclusions are provided.

## 2. IMU Requirements

This section presents the requirements that the developed *9* × *2* IMU is designed to meet. These requirements arise from the need of an IMU able to not only work as a datalogger but to also implement online classifiers. In consequence, the main requirement for the IMU is to be multi-functional: (a) this device should register inertial signals with a stable sampling frequency of 200 Hz; and (b) the microcontroller's firmware must be able to host online supervised learning algorithms. This main requirement supposes the overlapped execution of detection algorithms intertwined with the data capture processes, which hampers the implementation of a stable sampling frequency and the communication with storage units or wireless modules.

Regarding the usability requirements, a long autonomy and minimum consumption are two important premises. More specifically, since we are interested into monitoring movement, the IMU device is required to approximately last a whole day. This way, in case of several days of monitoring, the user could charge the sensor during sleep periods. Finally, the last usability concern is to be wearable, *i.e.*, smaller or similar in size to current smartphones.

With respect to inertial signal requirements, the most relevant one is to include the following triaxial inertial sensors: an accelerometer, a gyroscope and a magnetometer. The sensors sampling frequency could be enough with 40 Hz for measuring human movement [37]. However, the IMU is required to sample at a frequency up to 200 Hz, being this frequency adjustable.

Regarding the capabilities of implementing online classifiers, the IMU should include a Digital Signal Controller (DSC) in order to reduce the microcontroller's load of work and increase the autonomy. This module is able to reduce the time spent in the calculus performed by the supervised learning algorithms and, thus, enables the microcontroller to remain more time in low-consumption mode. The detection performed by the online classifiers must be shared with other devices, such a mobile phone, in order to, in the case of PD, provide audio cues through a wireless earphone during FoG episodes or to adjust the apomorphine doses administrated by an infusion pump. Therefore, the IMU is required to contain a wireless communication module. Finally, in respect of offline classifiers, one of the main goals of the IMU is to store inertial data during long periods to enable the training of supervised learning algorithms. Thus, a storage unit has to be included in the system.

In order to accomplish these requirements, a well organized firmware has to be designed. The firmware has to be flexible to allow the implementation of, for instance, machine learning or gait analysis algorithms.

## 3. Related Work

Many inertial measurement units have been utilized for research, health rehabilitation or sport tracking purposes. A detailed summary of the IMUs employed in these fields is presented in this section. Two main groups of IMUs are distinguished, on the one hand, those which only provide and store raw data from their inertial MEMS and, on the other hand, those which provide results of an implemented online classifier executed concurrently to the IMU's signal acquisition. However, none of the presented IMUs belong to both categories. Table 1 summarizes the most relevant IMUs presented in this section. In Section 4, where the *9* × *2* is detailed, a table which contains the same information as Table 1, but with the main features of the *9* × *2* has been included

The following IMUs form part of the first group, those which capture and store raw data by means of a peripheral storage unit or by a wire or wireless connection to a PC: MTw from Xsens (Enschede, The Netherlands) is a 10 axis wireless IMU, including accelerometer, gyroscope, magnetometer and a pressure sensor. Its dimensions are 34.5 × 57.8 × 14.5 mm^3^ and its weight, with the battery, is 27 g. Due to its reduced size and its 3.5 hours autonomy, when sampling at 120 Hz, Xsens is conceived to be used in short tests. This device does not have a storage unit, so the data is only received through radio-frequency. Among the different Xsens IMUs, an interesting development is MTi-G, which includes a GPS for outdoors tracking. Although this new peripheral affects its autonomy (its consumption is 610 mW and it would make the autonomy last less than an hour sampling at 120 Hz in a 1,130 mAh lithium-ion battery of 4.2V) and this IMU does not have neither a storage unit nor wireless module [38–40], a GPS increase the field applications. Another well-known IMU is Shimmer (Shimmer sensing, Dublin, Ireland), which is the acronym of Sensing Health with Intelligence, Modularity, Mobility, and Experimental Reusability. This sensor provides the same nine inertial signals obtained by the *9* × *2* IMU and it comprises two modules: the sensor/unit platform and the daughterboard wireless 9DoF Kinematic sensor [41]. In this IMU, a Bluetooth module and a storage unit are included. Its sampling frequency is programmable from 5Hz to 50 Hz with the three sensor systems, although, if only the accelerometer is used, a 100 Hz frequency rate is achieved. Autonomy is not reported when data are stored or wirelessly sent at maximum frequency. However, it is reported that Shimmer platform includes a 450 mAh battery [41,42]. KineO from Technoconcept (Mane, France) is another IMU used as a datalogger. With a sampling frequency of 100 Hz, and a triaxial accelerometer, gyroscope and magnetometer, the nine inertial signals are sent via Bluetooth to a computer, lasting up to 4 h under these conditions [43]. Another interesting inertial datalogger sensor is Physilog (EPFL, Lausanne, Switzerland), which is able to store inertial data from a triaxial accelerometer and a gyroscope at 200 Hz. It has a consumption of 71 mA, which means that given 1130 mAh battery, it has an autonomy of 15.9 h [44,45]. The Vitaport Activity Monitor (Temec, Kerkrade, The Netherlands) is another datalogger IMU which has been used to study and analyze Parkinson's Disease patients symptoms [46–48]. It has a sampling frequency of 32 Hz but a size of 90 × 45 × 15 mm^3^ and weight of 1.36 Kg with the data recorder. This is only suitable for data collection, as this is too obtrusive to be used in home environments. More examples of inertial sensors are the 3DM-GX1 (MicroStrain, Williston, VT, USA) and the UAV V3 (SparkFun, Boulder, CO, USA), which are development boards which have to be connected to a computer to capture inertial data [49,50]. These IMUs have in common the fact that they enable the gathering of data, although online classifiers cannot be efficiently implemented, in the case of evaluation boards, or not even implemented, in the rest of IMUs, since they are commercial devices whose microcontroller is not programmable.

The second group comprises IMUs that provide already processed information derived from inertial sensors. In this case, raw data are not accessible [6,8,51–56]. In this group, IMUs exclusively designed for analyzing specific activities, such physical activity or energy expenditure, are found. For example, IDEEA (MiniSun, Fresno, CA, USA) is a micro-processor unit composed of five satellite-dual-axis-wired accelerometers and a central unit that analyzes data and sends results through RS-232 or USB connections to a computer. The main unit size is 70 × 54 × 17 mm^3^ and it weights 59 g. The whole system records data at 32 Hz [51,52]. IDEEA is not a datalog device, as it is specifically designed to measure physical activity. Dynaport (McRoberts, The Hague, The Netherlands) is another physical activity sensor which records energy expenditure, among other features, with a sampling frequency of 100 Hz, during up to 72 h [8,53]. A very light sensor is activPAL (PAL Technologies, Glasgow, UK), which only weights 15 g and stores physical activity information during up to 7 days at 10 Hz of sampling frequency [6,54]. A very long-term monitoring sensor for physical activity is RT3 (Stayhealthy, Monrovia, CA, USA). This device is the biggest IMU from those mentioned before due to its battery, since it has a size of 97 × 109 × 51 mm^3^.

This sensor lasts about 21 days, but its sampling resolution is only 1 Hz and it only has an accelerometer. It is able to provide the signal magnitude or the values from each accelerometer axis [55,56]. These IMUs have in common the fact that they provide real-time processed information. However, as with the first group of IMUs, these devices cannot be used to implement other online classifiers since they are commercial products and their microcontrollers are not programmable.

## 4. *9* × *2* Hardware Architecture

According to the requirements presented in Section 2, a flexible IMU able to gather movement data up to 200 Hz and act as an online device to provide real-time information is needed. Sensors considered in order to satisfy these requirements are those with a bandwidth higher than 400 Hz (twice the maximum sampling frequency) and the sensors included (accelerometer, gyroscope and magnetometer) are integrated in such a way that they can be optionally disabled by the microcontroller's firmware to avoid unnecessary power consumption. Furthermore, storage and transmitting capabilities may be required, depending on the offline or online behavior. To this end, different regulators are added in order to able and disable different parts of the circuit, including the three sensors and the communications module. Finally, in order to increase the IMU's autonomy, the microcontroller's firmware is designed to be able to enter into a low-consumption mode when no action is executed.

In this section, the hardware system according to the design decisions detailed and it is summarized, compared to and contrasted with other IMUs according to the characteristics shown in Table 1. Then, the hardware architecture of the *9* × *2* IMU is described, such as the inertial sensors included, their calibration, the microcontroller which governs the system, the power management of the IMU and finally the communications module. Since this section is devoted to describe the IMU's architecture, Section 5 details its firmware design.

**Figure 1.**
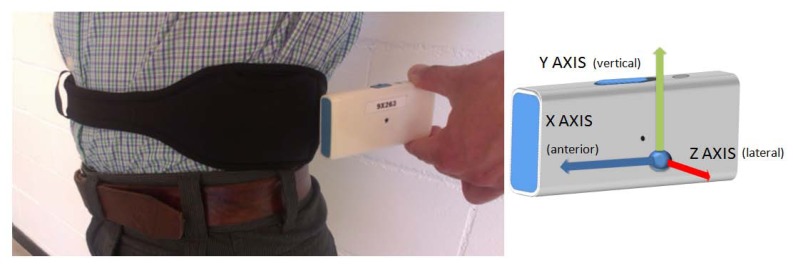
*9* × *2* sensor and its specially design neoprene belt.

The final design of the *9* × *2* IMU has a size of 77 × 37 × 21 mm^3^, and it weighs 78 g, a weight reduction of more than 30% in respect of a standard smartphone such as, for example, the HTC Desire X (137 g), Google Nexus 4 (139 g) or Apple I-Phone 5 (112 g). The *9* × *2* is a wearable platform with which users can perform their daily living activities normally with the purpose of getting inertial data in uncontrolled environments. This is, therefore, a non-invasive device, designed to have a long autonomy in order to capture data over long periods, without the need of recharging. Autonomy of the *9* × *2*, which is accurately detailed in Section 4.5, can last up to 36.81 h, and it has a power consumption of 113 mW (30.7 mA with a 3.7 V battery). These autonomy conditions given are for when the inertial data are written to a μSD card, the Bluetooth connection is not used and the sampling frequency is 200 Hz. Figure 1 shows a specifically designed neoprene belt with the IMU, its location when the sensor is used in the waist and its reference system.

**Figure 2.**
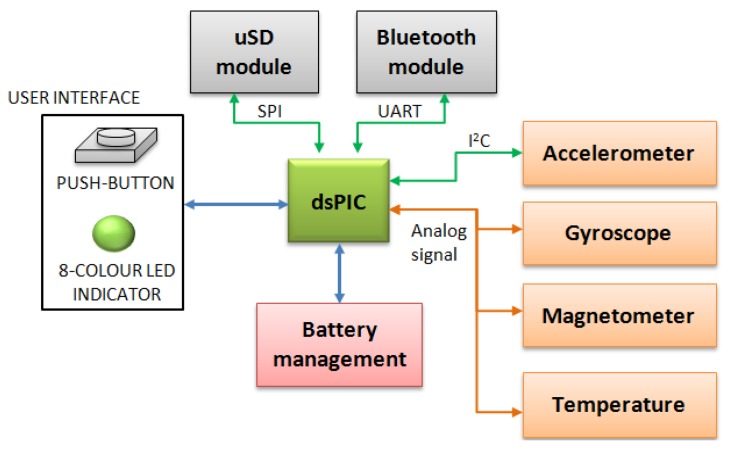
General block diagram.

Figure 2 shows the main structure of the *9* × *2* unit. As shown, the *9* × *2* is controlled by a dsPIC33F microcontroller, which has the main function of managing the different sub-systems which compose the system and to execute the implemented algorithms. The microcontroller also handles the power management and controls the user interface. The user interface consists of a push-button for basic instructions and a RGB LED which shows the *9* × *2* status, such as battery level, to the user. It also has a general switch which turns the system on and off.

The characteristics of the *9* × *2* are presented in Table 2 in a comparable way to those of the previously mentioned IMUs. The *9* × *2* is the only sensor which includes all the features except the GPS. Moreover, consumption at 200 Hz under similar use conditions is relevantly lower than that of the other datalogger IMUs. Size and weight are also acceptable, being smaller and lighter than a mobile phone.

### 4.1. Sensors

Inertial sensors are the main part of the *9* × *2* since they enable the movement analysis. In this section, the different sensors contained in the *9* × *2* and their characteristics are described.

#### 4.1.1. Accelerometer

According to the literature accelerometers are the most used inertial systems, since they are the most extended sensors for monitoring, assessing and analyzing human movements [6]. Consequently, the accelerometer is considered to be the most interesting sensor in the system and its measurements should be very accurate and stable. The LIS3LV02DQ (STMicroelectronics N.V., Geneva, Switzerland), from now on LIS3, was the accelerometer chosen since it provides a digital interface, includes the three axis accelerometer and the conditioning of the signal in the same package. This way, it is more robust to interferences as there are no electrical tracks through the PCB from the transducer. The LIS3 also incorporates a temperature sensor in order to re-calibrate the signal when necessary [57]. The LIS3 has 640 Hz of bandwidth which is sufficient to achieve a sampling frequency of 200 Hz. The sensor provides a programmable full scale of ±2 G or ±6 G. In the studies performed in CETpD, the sensor is used at waist, which is considered the most suitable location for measuring human movement according to some authors [14,15,58]. In this location, the range of accelerations measured from human movements reaches ±6 G [59]. Thus, this is the selected range for the LIS3. The sensor's sensitivity at ±6 G full range is 340 LSB/g or 2.9 mg/bit. Data are digitalized and sent to the microcontroller through an I^2^C bus at 1 Mbps.

#### 4.1.2. Gyroscope

Gyroscopes are interesting inertial sensors since the provide dynamic information through the angular speed. These measurements have been showed useful in the analysis of human movements such as gait [60], posture transitions [61] or falls [62]. Due to their widespread use in the video-games industry and mobile phone applications, they have become very low-cost devices.

The gyroscope used in the *9* × *2* has a range of ±2,000 °/s and a sensitivity of 0.5 mV/°/s. Two different gyroscopes, a biaxis and an uniaxial one, have been used to measure the angular rotation. The devices chosen are an IDG650 (Invensense, San Jose, CA, USA), a biaxial gyroscope that provides data for X and Y-axis [63], and an ISZ650 (Invensense) [64], which provides data for Z-axis. Their outputs are analog, which results in an important decrease of power consumption compared with the digitalization of the signal.

#### 4.1.3. Magnetometer

Magnetometers are widely used sensors for orientation purposes. However, their measurements are sensitive to inherent drifts and many external interferences, since they are very sensitive to magnetic changes such as those produced by nearby ferromagnetic objects. For this reason, magnetometers are commonly used as a complement to gyroscopes [65] and accelerometers [66,67] by means of fusion algorithms [68]. The magnetometer used in the *9* × *2* has a full scale of ±6 Gauss, and a sensitivity of 1 mv/V/Gauss. It is a dual system composed of a bi-axial magnetometer (HMC6042, Honeywell, Morristown, NJ, USA), which has the possibility to integrate the Z-axis by means of a second device (HMC1041Z, Honeywell) connected to it [69,70].

### 4.2. Calibration

Inertial sensors provide measurements affected by drifts and offsets. The characteristics of these changes are described in the datasheets provided by the manufacturers. In order to correct the measurements, a calibration process is necessary. The calibration performed on the *9* × *2* sensor relies on information provided in the data sheets, their application notes and a Least Squares technique described below [57,63,64,69,70].

The manufacturer of the LIS3LV02DQ provides information about an internal calibration curve, which compensates drifts that appear due to temperature. At any rate, according to sensor features, offsets of 100 mG (mili-gravity) can appear in any axis, non-linearity is 3% of the full scale, and the sensitivity change due to temperature is 0.025%/°C [57]. The gyroscopes used have a non-linearity of less than 1% of the full scale. They have a thermal drift around 10% near the limits of the operating temperature and mechanical intrinsic frequencies [63,64]. The HMC6042 and HMC1041Z magnetometers have a cross-axis sensitivity of ±0.2% FS/Gauss, a sensitivity to the temperature of −3,100 ppm/°C and an offset of ±10 ppm/°C. The magnetometers have a linearity error of 0.8% FS, an hysteresis error of 0.15% FS and a repeatability error of 0.11% FS [69,70].

A static calibration method has been used to adjust the inertial sensors, similarly to the method described by Giansanti *et al.* [71]. The calibration process consists in comparing the uncorrected measurements of the sensors with their actual values. Then, a Least Square method (LS) is applied to obtain the resulting calibration matrix *C_k_*. Once *C_k_* is obtained, the calibrated values (*x'_k_*, *y'_k_*, *z'_k_*) at any position are obtained by (*x'_k_*, *y'_k_*, *z'_k_*) = (*x_k_, y_k_, z_k_*)*C_k_*, where *x_k_, y_k_, z_k_* are the raw measurements.

The calibration matrix *C_k_* is defined as:

(1) $$C_{\text{k}}=\left(L_{k}\right){\left(S_{k}\right)}^{T}{\left[\left(S_{k}\right){\left(S_{k}\right)}^{T}\right]}^{-1}$$

where *k* denotes the sensor: *C*_a_, *L*_a_, *S*_a_ for the accelerometer, *C*_g_, *L*_g_, *S*_g_ for the gyroscope and *C*_m_, *L*_m_, *S*_m_ for the magnetometer. *L_k_* is a matrix containing the actual values extracted from known reference systems described below. Finally, *S_k_* is a matrix which contains the *9* × *2* raw measurements to be corrected.

**Figure 3.**
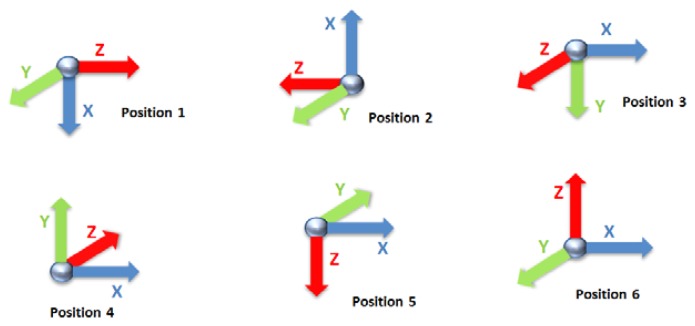
Accelerometer calibration positions.

The calibration of the accelerometer is based on using six positions for which the actual value is known, according to Figure 3. These positions correspond to the alignment of the three accelerometer axis with the gravity. During the alignments, 6 seconds of inertial data are recorded and filtered with a 2nd order Butterworth filter at 5 Hz.

With these six positions, 18 measurements corresponding to each axis are obtained. According to them, *S_a_* is filled as showed in Equation (2) and *L_a_* contains the gravity as shown in Equation (3). The LS method is then used to obtain the calibration matrix *C_a_* according to Equation (1):

(2) $$S_{a}=\left(\begin{array}{ccccc} x_{1} & x_{2} & x_{3}x_{4} & x_{5} & x_{6}\\ y_{1} & y_{2} & y_{3}y_{4} & y_{5} & y_{6}\\ z_{1} & z_{2} & z_{3}z_{4} & z_{5} & z_{6} \end{array}\right)$$

(3) $$L_{a}=\left(\begin{array}{ccccc} -G & G & 0\;0 & 0 & 0\\ 0 & 0 & -G\;G & 0 & 0\\ 0 & 0 & 0\;0 & -G & G \end{array}\right)$$

where *x_i_*, *y_i_*, *z_i_* are the measurements achieved at the position *i* according to Figure 3 and G = 9.81 m/s^2^.

In the case of gyroscopes, a Mti from Xsens Technologies [39] with a calibration certificate has been used in order to obtain actual angular velocity measurements in *L*_g_. The Xsens and the *9* × *2* are mounted on a turntable, which is driven by a DC motor powered with a QL355TP precision power supply from Thurlby Thandar Instruments (Huntingdon, United Kingdom) [72]. Matrices *L*_g_ and *S*_g_ are filled with the Xsens and *9* × *2* angular velocities, respectively, at the six positions of Figure 3.

Finally, the magnetometer system has been calibrated by means of comparing its output values *S*_m_ to the Earth's magnetic field, knowing that Earth's North and South magnetic poles are the maximum and minimum values the sensor has to be compared with. These values are well known at any place of the Earth through the National Ocean and Atmosphere Administration (NOAA), which has a tool that provides the Earth's magnetic field of a given location in the globe within 5 years (http://www.noaa.gov/). This tool is the gold–standard to calibrate magnetometers. Each measurement has been recorded during 6 seconds and compared to the actual values. The measurements of the Earth's magnetic field were taken in an area isolated from interfering elements (ferromagnetic elements) in order to obtain accurate measurements and avoid noise induced by these elements in the calibration measurements. *S*_m_ matrix is created by obtaining the maximum and minimum measurements of the sensor by turning 360° respect the Earth plane. These maximum and minimum value are known in the geographical point in which the experiment took place and, therefore, they are the reference *L*_m_.

### 4.3. Microcontroller

The whole system is controlled by the dsPIC33FJ128MC804 (Microchip, Chandler, AZ, USA), a microcontroller (MCU) which has an internal DSC. This MCU has a modified Harvard architecture, with 16-bits of data path, and 24-bit wide for instructions. It has 128 KB of Flash Program Memory and 16 KB of RAM. Digital communications peripherals are UART, SPI and I^2^C, which controls Bluetooth, μSD card and the accelerometer, respectively. It also has one ADC with nine input channels of 10 bits, which is enough to capture the seven analog signals (three × gyroscope, three × magnetometer, one × temperature).

The MCU has a Real-Time Clock Counter (RTCC) engine to calibrate the RTCC up to an error of ±2.64 s a month according to its datasheet. However, and due to intrinsic fabrication processes, the crystal frequency can be different in each device and the RTCC must be accurately calibrated. A Universal Frequency Counter (53132A, Agilent, Santa Clara, CA, USA) has been used for this purpose [73]. A curve of the frequency drift depending on the temperature is given by the crystal manufacturer in order to adjust RTCC (Abracon, Rancho Santa Margarita, CA, USA).

The MCU's frequency is 80 Mhz while the CPU is able to work at 40 MIPS. This frequency can be tuned with a register designed for this purpose. The microcontroller's frequency (FCY) has been also calibrated with the universal frequency counter 53132A. Thus, the tuning register has been modified to achieve the most correct frequency. Furthermore, considering the frequency drifts due to temperature, a correction to the frequency is applied according to the curve provided by the dsPIC33F data sheet [74] and measurements from the sensor temperature included in the *9* × *2*.

The *9* × *2* corrects the RTCC measurements and its tuning register values according to the current temperature every minute. Consequently, part of the curve provided by the manufacturers is implemented and the correction factors for the current temperature are applied. In our case, the curve comprised between 0 and 60 degrees is implemented.

The dsPIC has a Direct Memory Access (DMA) system which allows transferring data from peripherals to buffers and data stored in the RAM with a minimum action of the MCU's. DMA and the different power-saving modes permits the *9* × *2* to be a very low-consumption system. The dsPIC allows three main power-saving modes: Run mode, Idle mode and Sleep mode. Run mode is the mode in which CPU and peripherals are working normally. Idle mode offers the possibility to sleep the MCU except a selected peripheral. Then, the system clock remains active, but no instructions are executed. An interruption will awake the MCU when it is necessary, reducing its consumption from 63 mA to 34 mA (disabling all the MCU peripherals) [74]. During the sleep mode, only external clock inputs or external interrupts are allowed to awake the MCU. This mode drastically reduces the consumption to 10 μA. Peripherals can also be disabled depending on the use, avoiding unnecessary consumptions.

### 4.4. Power Management

**Figure 4.**
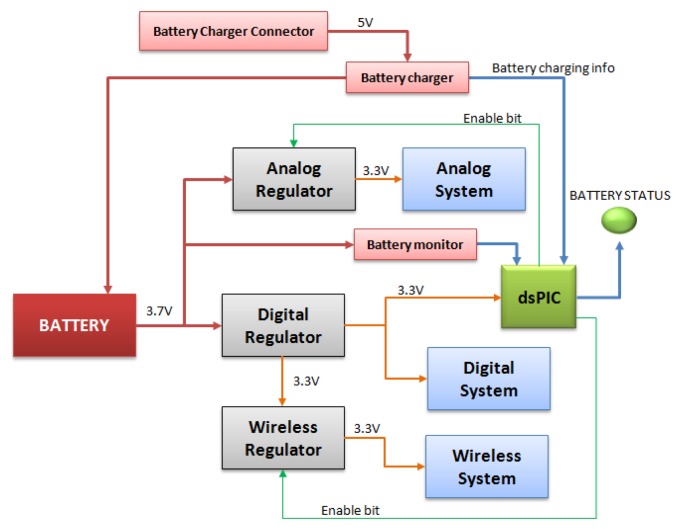
Power management block diagram.

The *9* × *2* includes a battery management system which enables saving energy. As shown in Figure 4, there are three low-dropout (LDO) regulators which enable not feeding the selected parts of the PCB. The first LDO regulator supplies the MCU, which manages the other LDO regulators. The other two LDO regulators supply the analog circuit and the wireless circuit.

The system is supplied by a 1,130 mAh lithium-ion battery. The autonomy of the system is approximately 36.8 h at a sampling frequency of 200 Hz and while continuously storing the data provided by a triaxial accelerometer, gyroscope, magnetometer, the temperature values and the battery status. The Bluetooth system is disabled under these conditions. However, the Bluetooth system allows the user to send data collected to a computer at a frequency between 1 Hz and 200 Hz. When the system sends at 200 Hz via Bluetooth, the autonomy decreases from 36.81 h to 18 h, since Bluetooth's consumption is higher than that of the μSD Card during the storage process. This autonomy is sufficient to cover data captures of all the current projects the *9* × *2* is participating. According to the requirements of the research projects, the IMU's usage is suggested to be similar to that of a mobile phone. Then, the device should be charged a maximum of once per day, and normally during the night. In this manner, 18 h or 36 h are sufficient to cover both aspects (data capture and real-time evaluation).

The system incorporates a battery charger and a battery monitor. The battery monitor indicates to the user the current state through a RGB-LED. When the battery is very low, an interruption is produced in the MCU and the system automatically closes all its peripherals and communications and enters in the Sleep mode.

In order to reduce consumption, the firmware can enable and disable the Analog and Wireless Regulator when necessary. The system remains in operating mode only when data need to be captured, data are needed to be sent or when an algorithm is being executed.

An accurate analysis of the *9* × *2′*s autonomy has been performed in different operating modes. The *9* × *2* can either work with the Bluetooth in order to receive commands from a terminal, or can send the inertial data or algorithm results to this terminal. However, the *9* × *2* can operate without the Bluetooth module, or switching it on only when necessary. These cases are used when a data capture is performed, or when an online classifier is running and results are stored in the μSD. These cases are reported in Table 3. These autonomies have been measured after the system has been running over 2 min and they have been calculated taking into account the battery capacity (1,130 mAh) and the average consumption in a period of 10 min. This process has been repeated five times in order to take accurate measurements. Results shown in Table 3 are the mean and deviation of these five measurements. These results show that the online classifier behavior has an autonomy of 41 h at 40 Hz, the highest autonomy after the datalog behavior without Bluetooth, which is 45 h. When Bluetooth is used, autonomy decreases to about half of that duration.

### 4.5. Communication Modules

According to the requirements detailed in Section 2, the IMU has to wirelessly communicate with, at least, a mobile phone to share the output of the algorithms. Current mobile phones only offer Bluetooth as a low consumption communication solution with a high bandwidth and rate transfer. Thus, the *9* × *2* is forced to use a Bluetooth module in order to satisfy this requirement. Bluetooth works at 115,200 bps in the *9* × *2* system and its consumption is 31.5 mA according to its specifications [75]. Furthermore, in order to behave as a datalog system, the *9* × *2* also includes a μSD card socket. Bluetooth enables the wireless reception of all data captured by the *9* × *2* while the μSD card permits storing data without maintaining a continuous wireless communication.

The μSD card socket is useful to develop offline classifiers since it is used to build databases in order to elaborate future algorithms. Its main advantage is that it does not need to be connected to any computer while getting data. In the REMPARK project, patients who form part of the collection of inertial data perform their normal daily living activities with the sensor attached to them while the sensor is collecting the inertial data and storing it into the μSD card [34,35,76]. Then, this data is processed offline and classifiers are designed.

## 5. Firmware of the Inertial System

Firmware is the most relevant part of the system since its main functions are: (1) manage the peripherals in order to guarantee an accurate and stable sampling frequency; (2) supervise the correct operation of communications; (3) control the processes to store the inertial data captured before sending it to a peripheral while it controls battery status and user interface; and (4) execute online classifiers. One of the main requirements of the firmware is to optimize the consumption. To this end, a specific firmware has been developed and real-time OS's are avoided to optimize the consumption and code memory usage. Moreover, the MCU properly fluctuates among different work modes (Run, Idle, Sleep). In this section, the main firmware process and the state machine associated that manages the firmware are presented, as well as the internal processes that take a relevant role in the *9* × *2* firmware.

### 5.1. Main Process

A major focus for the *9* × *2* was to obtain a firmware in which the MCU only works in run mode when necessary and, thus, remained in a low-consumption mode most of the time. In order to achieve this premise, the different processes involved in the data acquisition, the communications, the battery management and the online classifiers were prioritized.

**Figure 5.**
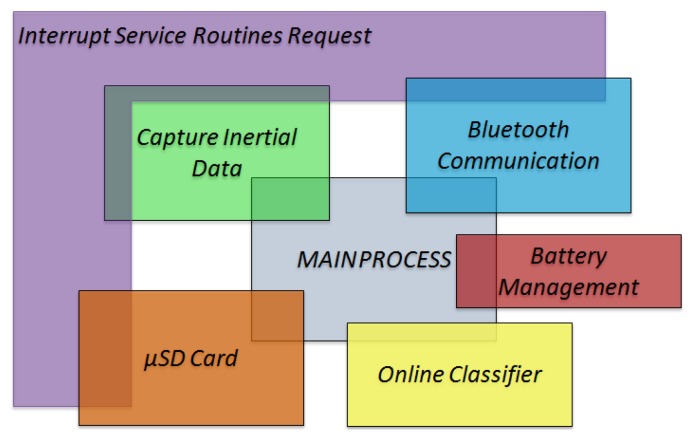
Firmware's processes interaction.

Figure 5 presents a diagram that describes how these independent processes interact with the main process and their relation with the interruptions. The main process initializes the other five processes. Some processes are only executed after an interrupt request. For instance, the process of capturing inertial data is executed immediately after an interrupt request of the data acquisition timer. Bluetooth and μSD Card processes are also executed only when an interrupt request is produced. In the case of μSD Card, when the acquisition buffer is full, a DMA interrupt is launched which starts the data storage. In the Bluetooth case, bluetooth reception interrupts launch the corresponding process when an external command is received. Moreover, timer interrupts also launch the Bluetooth process in order to send data, such as algorithm's output or raw data. Battery management and online classifiers are executed within the main process both after data are captured and before the MCU enters Idle mode.

The main process comprises of an initialization phase, in which the peripherals are configured and associated to a DMA channel. Peripheral interrupts are enabled and a priority is given to each peripheral. The process with most priority on the system is the timer which controls the sensors samples acquisition. After this process, data collected are stored in RAM. These data can be analyzed and sent within a specific frame to a DMA channel. The firmware allows sending all inertial data through Bluetooth or through μSD card. However, if an algorithm is executed, data sent through communication modules may not be the raw data but the results of the algorithm. The latter case enables an energy saving, since communication modules are the most consuming parts of the *9* × *2*.

Online classifiers and battery management are processes with the least priority. They are only executed when the rest of processes allow free clock cycles in the MCU. However, algorithms can be associated to interrupts depending on the user's design, so they can be executed, for example, right after an interrupt request of the capturing data process for filtering purposes.

The battery management process consists of controlling the battery monitor. The MCU informs on the battery status through the user interface. However, if a very-low battery signal is received, the system closes all the communications and then sets the MCU in sleep mode. This method allows saving data files, and avoids losing information if the MCU suddenly switches off due to the low voltage. An external interruption has been configured to interrupt all processes when the user connects the *9* × *2* to charge the battery, since it is considered that it is not necessary to keep the system awake when the battery is being charged.

**Figure 6.**
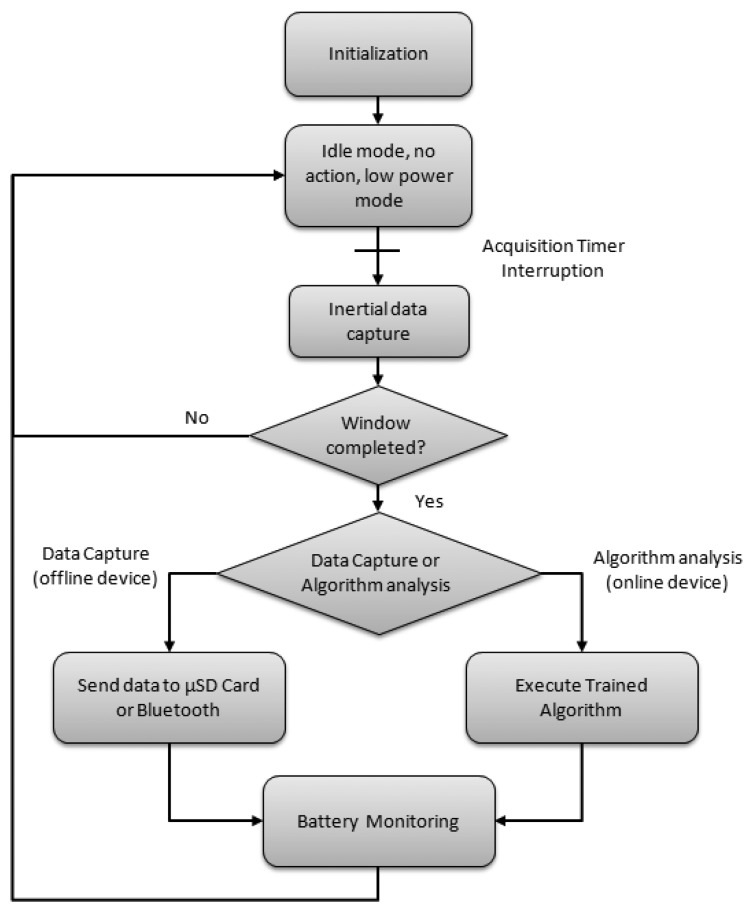
Firmware sequence.

The algorithmic task is executed into two different parts. On the one hand, in the interrupt request function associated to the data acquisition some simple computations are performed, such as the signal filtering. On the other hand, since online classifiers are usually based on signal windows [77,78], once a window is filled with inertial data, the computations take place. When the system is programmed only for logging data, no algorithm is executed and the system works in the Run mode during the data capture process or communication modules interruptions are requested. Figure 6 shows the basic structure of the firmware summarizing its dual behavior.

In order to check *9* × *2′*s performance, two groups of tests have been performed, the first one writing continuously to a μSD without a Bluetooth connection, and the second one sending inertial data continuously through Bluetooth. The system has been tested while sampling at 200 Hz and the tests have been executed 10 times in order to achieve accurate data. On the one hand, the MCU remains an average of the 95.46% ± 2.0266% of the total process time in Idle mode when data is being sent to the Bluetooth module with a sampling frequency of 200 Hz. On the other hand, the CPU remains in the Idle state an average of 99.2% ± 0.0146% of the total process time when the device is writing to a μSD card.

### 5.2. Data Capturing Process

The main priority of the MCU is to guarantee a stable and accurate sampling frequency. Thus, the other processes should not have more priority than the data acquisition and they should be halted when an interruption for acquiring data is requested.

In order to achieve it, the sampling acquisition instants are controlled by a timer which depends directly on the MCU's frequency oscillator, which was calibrated to achieve an accurate frequency. Sampling frequency is 200 Hz by default, but the user can modify this frequency. Once the data acquisition timer interrupt is requested, the ADC module is called to collect analog data and the accelerometer data is captured through I^2^C. Then, these data are arranged and stored in RAM ready to be sent through SPI to the μSD card or through UART to the Bluetooth module. The data sent is composed of the inertial data (accelerometer, gyroscope and magnetometer), temperature, time taken to collect data, and times the last variable was sent, battery status and device identification. The data sent can be changed to, for instance, the RTCC register values, *i.e.*, the current time. Data are first stored in a dedicated RAM, which is updated for each captured sample. Algorithms which work with inertial information use data windows. Therefore, data corresponding to several seconds are saved. More concretely, 5 KB of RAM are dedicated to store inertial data.

### 5.3. Bluetooth Connection

Bluetooth module performs two main functions in the *9* × *2* unit. The first one is to receive instructions from an external system (PC, tablet, mobile phone). The second one is to send frames built (described in Section 5.2) to the external systems. There is another extra option, which is getting data from another complementary inertial device. The MCU then sorts these data in the frame and stores it in the μSD.

At the beginning of the program the system checks if a μSD card is connected, then the system waits for instructions from an external system. In the data logging performed in the REMPARK project, for example, a PC sends the current time via Bluetooth to the *9* × *2*, which is stored in the RTCC to maintain the current time. Another application of Bluetooth is, for instance, to receive the sampling frequency through instructions before collecting inertial data, so the *9* × *2* begins to capture and store data in a μSD card. Through this system, the IMU can be paused if necessary in order to avoid getting unnecessary data. Finally, in the PD application, results of the algorithms are sent to a mobile phone.

### 5.4. μSD Communication

SD flash memory is essential to store inertial data. This module allows capturing data without a wireless connection. μSD Cards used are 2 GB size with a FAT16 system file. It allows storing data continuously during 3 days and 19 h sampling at 200 Hz, or 19 days and 10 h sampling at 40 Hz. Since the *9* × *2* uses FAT16, frames are built under this file system and they contain 32 bytes. A cluster in a FAT16 file system is 16 KB, which contains 32 sectors of 512 bytes each one. Each sector contains 16 inertial data frames. Processes to manage clusters and sectors are totally performed by a specific state machine which is only executed when necessary in order to avoid disturbing the main process. The MCU only controls whether a sector is full, in which case it copies RAM data to DMA memory and sends an order to the DMA to start writing data through the SPI bus. Since SD cards are flash memory devices, the process of writing a block through SPI to a SD card may last up to 250 ms. When a new cluster or sector has to be selected, the MCU sends new instructions to the SD card, which needs extra time to be executed and so new ranges of memory can be written [79]. During this time, MCUs RAM memory has to store data which is sampled every 5 ms for 200 Hz sampling frequency. Five KB of RAM memory is dedicated to store inertial data; since each frame is 32 bytes long 160 frames can be stored. At a frequency of 200 Hz, the buffer is filled in 800 ms. This time period is sufficient to guarantee that no data are lost since the SD card takes up to 250 ms in order to write a block.

### 5.5. Algorithmic Process

In order to analyze the sensors' measurements in real time, data is commonly windowed in short periods [77,78]. Moreover, algorithms usually overlap these windows at 50% in order to avoid losing event information [80,81]. In our system, these principles are also followed and these data windows are stored in the MCU's RAM, as these inertial data are continuously being updated along time. Flash memory contains all constants and parameters calculated in the off-line process that are needed to be used in the real time processing.

Among the processes that the MCU manages, the process dedicated to execute the online classifier implemented in the MCU, along with battery status process, is the process with less priority. An algorithm process can be a long process. Since one of the premises is to guarantee accuracy in sampling frequency and the correct transfer of data between modules, the algorithm may be interrupted for any of these processes and, then, continue. Although latency time of an algorithm is important, in terms of body movement, few microseconds during which the algorithm's process can be delayed are not relevant.

**Figure 7.**
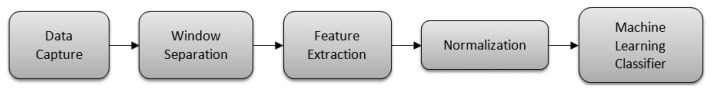
Classical classification method.

Memory management in the online classifiers is also a relevant aspect. On the one hand, when data are stored in the μSD card, windows of 512 bytes are recorded to be sent to μSD. On the other hand, signal windows to be analyzed at the algorithm are also built. Memory among windows is shared. A window counter will alert the MCU when a window has been filled with samples and, then, it is ready to be analyzed. Then, signals are filtered and features to be analyzed are selected and extracted, according to Figure 7. Then, threshold based trees, neural networks, Support Vector Machines or other classifiers might be executed. Results of the algorithms can be sent through Bluetooth or stored in the μSD card for debugging purposes. These results can also be analyzed to diagnose some disease and then sent the diagnosis results to any other module. Finally, the system enters in Idle mode in order to save energy. In the online development of the sensor device for the real-time treatment of PD, the Digital Signal Controller motor has been used to calculate array operations in few cycles. More details can be found in the next subsection.

### 5.6. Online Classifier for Gait Detection in PD

The *9* × *2* was used in a medical application, as part of HELP project, in which it provided the information to a clinician to remotely regulate an apomorphine pump. Doses administrated by the pump were increased when patients were in OFF state, a motor state mainly associated with a lack of medication. This OFF motor state was determined through a gait analysis performed in real-time by the *9* × *2* [21]. As a first step of this gait analysis, the *9* × *2* establishes if patient walks. To this end, artificial intelligence algorithms were introduced within the MCU in order to behave as an online device. Specifically, Short Time Fourier Transform and Support Vector Machines have been implemented in the MCU in order to lighten its work load. The scheme of this gait detector implementation followed a classical classification problem approach, which can be divided into different parts as shown on Figure 7: data capture, window separation, feature extraction and machine learning classification [82].

A pre-requisite to perform the gait analysis in the HELP project was to identify when a PD patient was walking or not by means of the *9* × *2* located at the waist. Hence, a database of movement signals was gathered in which inertial data from different PD volunteer patients executing different activities were captured. This database was used to train a SVM in order to distinguish walking from not walking periods [21]. It was taken into account that the model had to be executed within a MCU and the number of support vectors had to be minimized. A SVM model with 66 support vectors was finally obtained and implemented within the MCU. Following Figure 7, the online device first captured inertial data and then, when the data window was completed, features belonging to this window were calculated. In this concrete problem, two features belonging to the data window power spectral density are extracted by means of FFT. Then, these calculated features are normalized in order to be comparable. The normalized data are the input for the SVM model which gives the output of the problem, in this case, whether the person is walking or not.

The time that each of the phases described in Figure 7 consumed in the SVM implementation are described in Table 4. These time measurements were obtained through an oscilloscope connected to the *9* × *2* by activating and deactivating digital outputs of the MCU when each one of the stages described in Figure 7 started or finished. The windows size of the SVM was 3.2 s length and the frequency sampling was reduced to 40 Hz. Windows were 50% overlapped and each window contained 128 samples. As Table 4 shows, every 1.6 s the MCU was awoken to begin calculation, then, a total of 44.86 ms are needed to calculate feature extraction, data normalization and SVM classification. This means the MCU remains in Idle mode or processing the other processes (Data Capture, Bluetooth, μSD Card, Battery Management) during 97.2% of the total available time.

## 6. Conclusions

An inertial measurement unit for long-term monitoring called the *9* × *2* has been designed. This IMU has already been used in previous developments which aimed to detect PD symptoms and to treat them in real-time [27,28,33,35,76]. Currently, the *9* × *2* is being used to collect movement signals in four different countries from 90 PD patients under the auspices of the REMPARK project [34,35]. The second part of this project consists in designing intelligent algorithms capable of monitoring different PD symptoms (freezing of gait, dyskinesia, tremor, bradykinesia, falls and gait parameters). Moreover, a new sensor based on the *9* × *2′*s hardware and firmware is also being validated as part of a fall detector system in a pilot test with 200 individuals [36]. Finally, the *9* × *2* has also been employed in research projects [81,83–86].

The IMU presented contains a triaxial accelerometer, gyroscope, and magnetometer. It also includes a temperature sensor. The *9* × *2* has a power management module which contains a battery charger and a battery monitor that provides information of the battery status to the user. The sensor is totally wearable being smaller than a mobile phone and suitable to be worn on the body with, for instance, the specifically developed neoprene belt shown in Figure 1.

This system has a programmable sampling frequency from 1 Hz to 200 Hz, which is enough to analyze human movements [37]. Sampling at 200 Hz, and compared to other commercial sensors, it has the longest autonomy compared to other inertial data loggers. The *9* × *2* is, moreover, programmable to execute online classifiers. The time which the CPU allows for executing algorithms is about the 99.2% of the total time when raw data is stored at 200 Hz in the μSD card, or 95.46% if raw data is sent through Bluetooth at 200 Hz. The clock frequency of the *9* × *2* has been fully calibrated with a specific device (a 53132A universal frequency counter), and a specific method has been designed to calibrate the inertial sensors. The system has been designed with the possibility to disable specific hardware. The firmware enables the peripherals of the MCU to be disabled to reduce consumption.

The IMU offers the possibility to send inertial data to external devices and the possibility to store it in a μSD card with almost 4 days of data capacity. However, the *9* × *2* lasts for 36.8 h while continuously storing data without recharging the device. It also includes the possibility to import external data such as another inertial device to compare two different signal sources in real time.

Online classifiers have already been tested in, for instance, a pilot study in which an apomorphine pump was regulated according to the symptoms detected by the *9* × *2′*s algorithms [28]. In this case, a Short Time Fourier Transform and Support Vector Machine algorithm were computed in real-time and were optimized through a Digital Signal Controller, speeding up the algorithm and saving energy.

The designed IMU is a specific and useful tool for research which opens wide possibilities in the field of human movement analysis research. Moreover, it is a very flexible system capable of gathering signal databases and executing algorithms under many conditions, such as selecting a sampling frequency, selection of sensors to use (accelerometer, gyroscope and magnetometer), storing data in a SD or sending raw data through Bluetooth. Finally, the *9* × *2* enables the development and implementation of online classifiers, so that their outputs might be stored or sent.

## Figures and Tables

**Table 1.** Commercial Inertial Measurement Units comparison.

**Name**	**Manufacturer**	**Sample freq/Hz**	**Autonomy info**	**Size/mm^3^**	**Weight/g**	**Raw data datalog capacity**	**Storage unit**	**Wireless**	**Acc**	**Gyro**	**Magn**	**GPS**
Mtw	Xsens	120	3.5 h	34.5 × 58 × 4.5	27	Yes	No	Yes	Yes	Yes	Yes	No
Mti-G	Xsens	120	610 mW	58 × 58 × 28	68	Yes	No	No	Yes	Yes	Yes	Yes
MainUnit + Wireless DoF	Shimmer	50	450 mAh	53 × 32 × 25	22	Yes	Yes	Yes	Yes	Yes	Yes	No
KineO	Technoconcept	100	4 h	49 × 38 × 19	25	Yes	No	Yes	Yes	Yes	Yes	No
Physilog 3	EPFL	200	71 mA	50 × 40 × 16	36	Yes	Yes	No	Yes	Yes	No	No
3DM-GX1	MicroStrain	350	65 mA	64 × 90 × 25	74.6	Yes	No	No	Yes	Yes	Yes	No
UAV V3	SparkFun	40	420 mW	38 × 70 × 25	34	Yes	No	No	Yes	Yes	No	Yes
IDEEA	MiniSun	32	48 h	70 × 54 × 17	59	No	No	No	Yes	No	No	No
Vitaport activity monitor	Temec	32	12 h	90 × 45 × 15	1360	No	Yes	No	Yes	No	No	No
DynaPort	mcroberts	100	72 h	64 × 62 × 13	78	No	Yes	No	Yes	No	No	No
activPAL	paltechnologies	10	7 days	53 × 35 × 7	15	No	No	No	Yes	No	No	No
RT3	Stay healthy	1	21 days	97 × 109 × 51	65	No	Yes	No	Yes	No	No	No

**Table 2.** *9* × *2* features.

**Name**	**Manufacturer**	**Sample freq\Hz**	**Autonomy info**	**Size/mm^3^**	**Weight/g**	**Raw data datalog capacity**	**Storage unit**	**Wireless**	**Acc**	**Gyro**	**Magn**	**GPS**
9 × 2	CETpD	200	36.8 h	77 × 37 × 21	78	Yes	Yes	Yes	Yes	Yes	Yes	No

**Table 3.** Operating mode autonomies.

**Online classifier without Bluetooth**										
Sampling Frequency/Hz	40		50		100		150		200	
				
Autonomy/h	41.09	±0.2	40.07	±0.2	35.99	±0.4	31.22	±0.15	27.63	±0.1

**Data capture with μSD and without Bluetooth**										

Sampling Frequency/Hz	40		50		100		150		200	
				
Autonomy/h	45.56	±0.2	44.84	±0.25	42.97	±0.1	40.21	±0.1	36.81	±0.2

**Sending continuous data through Bluetooth**										

Sampling Frequency/Hz	40		50		100		150		200	
				
Autonomy/h	19.12	±0.35	18.96	±0.2	18.80	±0.1	18.28	±0.0	17.94	±0.2

**Data Capture for offline classifier with Bluetooth connection**										

Sampling Frequency/Hz	40		50		100		150		200	
				
Autonomy/h	20.18	±0.45	19.82	±0.3	19.15	±0.4	18.83	±0.3	18.23	±0.25

**Table 4.** MCU's computation times.

**Events**	**Time duration of events**
Computation start	Every window (1.6 s)
Feature Extraction	35.07 ms
Data Normalization	0.04 ms
SVM classification	9.75 ms
Total computation time	44.86 ms
